# Systemic toxicity of CAR-T therapy and potential monitoring indicators for toxicity prevention

**DOI:** 10.3389/fimmu.2024.1422591

**Published:** 2024-08-26

**Authors:** Jingxian Li, Huiguang Chen, Chaoping Xu, Mengci Hu, Jiangping Li, Wei Chang

**Affiliations:** ^1^ Institute of Infection, Immunology and Tumor Microenvironment, School of Medicine, Wuhan University of Science and Technology, Wuhan, China; ^2^ Department of Hematology, Puren Hospital Affiliated to Wuhan University of Science and Technology, Wuhan, China; ^3^ Department of Blood Transfusion, Puren Hospital Affiliated to Wuhan University of Science and Technology, Wuhan, China

**Keywords:** chimeric antigen receptor T cell, hematologic malignancy, adverse events, biomarkers, toxicity, monitoring indicators

## Abstract

Malignant tumors of the hematologic system have a high degree of malignancy and high mortality rates. Chimeric antigen receptor T cell (CAR-T) therapy has become an important option for patients with relapsed/refractory tumors, showing astonishing therapeutic effects and thus, it has brought new hope to the treatment of malignant tumors of the hematologic system. Despite the significant therapeutic effects of CAR-T, its toxic reactions, such as Cytokine Release Syndrome (CRS) and Immune Effector Cell-Associated Neurotoxicity Syndrome (ICANS), cannot be ignored since they can cause damage to multiple systems, including the cardiovascular system. We summarize biomarkers related to prediction, diagnosis, therapeutic efficacy, and prognosis, further exploring potential monitoring indicators for toxicity prevention. This review aims to summarize the effects of CAR-T therapy on the cardiovascular, hematologic, and nervous systems, as well as potential biomarkers, and to explore potential monitoring indicators for preventing toxicity, thereby providing references for clinical regulation and assessment of therapeutic effects.

## 1 Introduction

Malignant hematologic cancers, including leukemia, lymphoma, and multiple myeloma, are a group of hematopoietic diseases characterized by high malignancy, complex treatment, and poor prognosis. These cancers represent a significant challenge in clinical settings due to their highly aggressive nature and the difficulties associated with their management. The primary clinical treatments include high-dose radiotherapy and chemotherapy, or even bone marrow transplantation (1). Despite advancements in treatment, hematologic malignancies have long been a significant public health issue due to their limited responsiveness to conventional chemotherapy and high recurrence rates. Despite systematic chemoradiotherapy, over half of the young adult patients and about 90% of elderly patients with acute myeloid leukemia (AML) still die from disease progression (2). An estimated 40% of lymphoma patients face relapses or treatment-resistant disease post-therapy (3); virtually all individuals with Multiple Myeloma (MM) are bound to experience a relapse (4). In recent years, cancer immunotherapy has emerged as a research focus, with CAR-T therapy proving to be an effective treatment for hematologic malignancies. The principle of Chimeric Antigen Receptor T (CAR-T) therapy involves using gene-editing technology to insert sequences of CAR that can target tumor surface antigens into T cells. After expanding these cells *in vitro*, they are reintroduced into the patient to specifically target and kill tumor cells (5). Clinically, CAR-T therapy is primarily used to treat relapsed/refractory B-cell malignancies, including Acute Lymphoblastic Leukemia (ALL), Diffuse Large B Cell Lymphoma (DLBCL), Follicular Lymphoma (FL), MM, and Mantle Cell Lymphoma (MCL) (6). Its application to other hematologic malignancies such as AML (7) and T-cell acute lymphoblastic leukemia/lymphoma (T-ALL/LBL) (8) is also under research. This therapeutic approach has fundamentally altered cancer treatment paradigms. Data from the World Health Organization’s International Agency for Research on Cancer (IARC) in 2023 shows that the five-year survival rates for myeloma and non-Hodgkin’s lymphoma have improved from 32% and 56% in 1995-1997 to 58% and 74% in 2012-2018 (9), marking significant clinical achievements.

**Figure 1:**
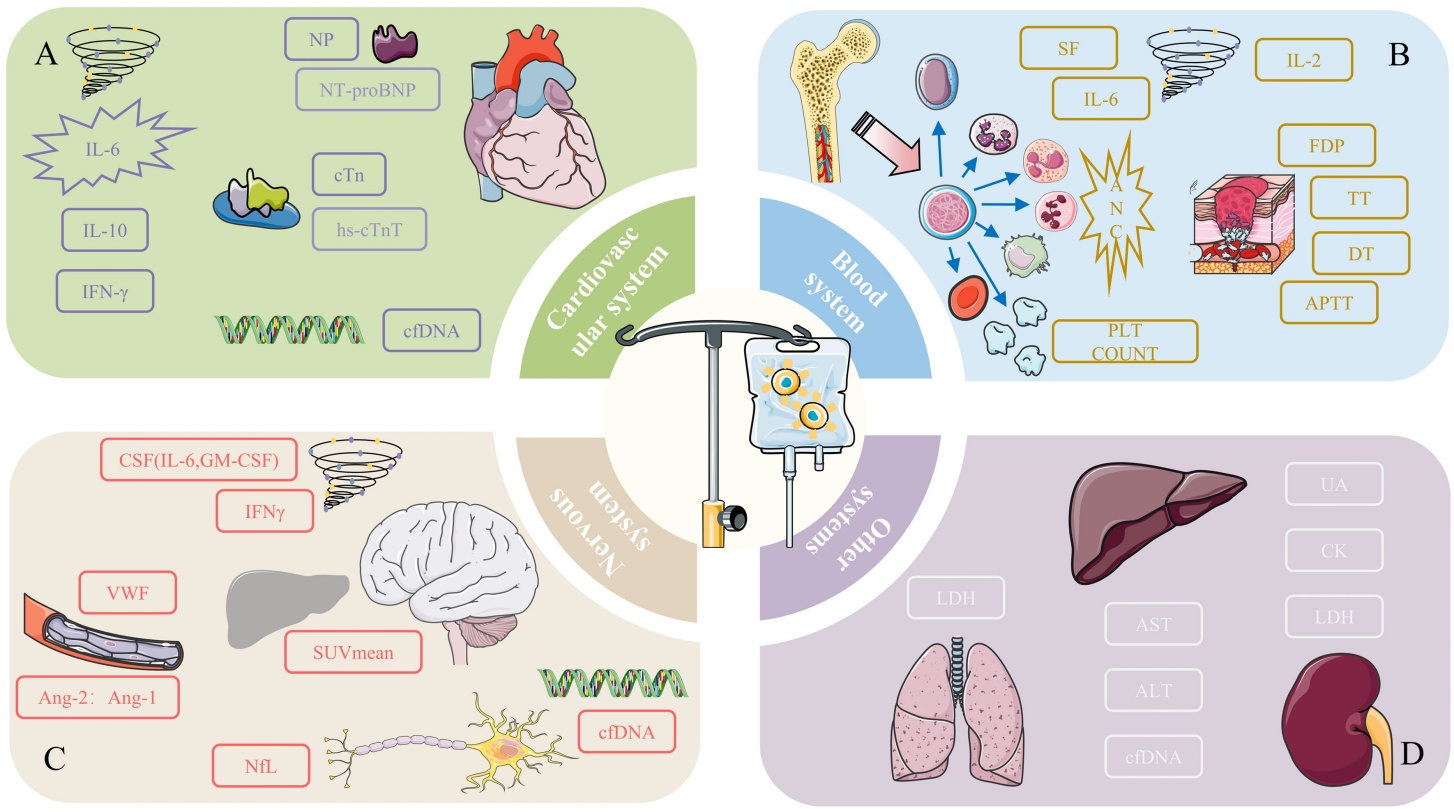
Potential monitoring indicators for preventive toxicity of **(A)** cardiovascular system, **(B)** blood system, **(C)** nervous system, and **(D)** other systems. *(A) For the cardiovascular system, the designed content in the figure may be the monitoring indicators we can focus on. High concentrations and peak levels of related cytokines (IL-6, IL-10, IFN-γ) are highly associated with adverse cardiovascular events. The peak concentrations of special proteins NT-proBNP and cTn hs-cTnT from the NP family are positively correlated with the severity of cardiotoxicity. Specific cfDNA can be used to predict the occurrence of cardiotoxicity. **(B)** Blood cell counts are particularly important in the management of hematologic toxicity, especially neutrophil counts. Coagulation-related markers such as FDP, TT, DT, and APTT are used to monitor coagulation disorders after CAR-T therapy. Cytokines (SF, IL-6, IL-2) are also potential monitoring indicators. **(C)** The cytokines that need special attention in the nervous system are IL-6, GM-CSF, and IFN-γ. Vascular-related active factors such as VWF and the Ang-2: Ang-1 ratio are noteworthy monitoring indicators. NfL, related to axonal injury, is a unique indicator of neurotoxicity. cfDNA can be used to predict the occurrence of toxicity, and the SUVmean of [18F] FDG PET in relevant organs can serve as an indicator of severity. **(D)** Due to the limited research involving other systems, only potential biomarkers and detection indicators are listed. Their specific roles need further research for confirmation.

Despite the remarkable therapeutic effects of CAR-T, its toxicity reactions cannot be ignored. The most common severe toxicity reaction associated with CAR-T therapy is Cytokine Release Syndrome (CRS). CRS occurs when a large number of lymphocytes are immunologically activated and CAR-T cells kill tumor cells, leading to the release of large amounts of cellular content, resulting in a cytokine storm-induced systemic inflammatory response. Main symptoms include fever, hypertension, and anemia (10). Immune Effector Cell-Associated Neurotoxicity Syndrome (ICANS), mediated by immune effector cells, is the second most common severe toxicity reaction in CAR-T cell therapy. Elevated serum cytokines and activated endothelial cells may lead to a cascade reaction, amplifying blood-brain barrier permeability, allowing high systemic concentrations of cytokines to diffuse and transport T cells to the central nervous system. The main common symptoms include tremors, writing disorders, mild speech difficulties (especially naming objects), and certain consciousness disorders, which can also manifest as some rare but more severe symptoms include ataxia, delirium, seizures, and cerebral edema (11, 12). Although the two classic CAR-T treatment toxic reactions have been thoroughly studied and reported, there is currently no systematic evaluation of toxicity in various systems. To achieve the reduction of CAR-T treatment toxicity within a controllable range while ensuring its effectiveness in killing tumor cells and effectively predicting patient prognosis, research on biomarkers for CAR-T cell therapy, as a “living drug” treatment, is particularly important. In this review, we discuss the potential toxic effects of CAR-T cell therapy on various systems, especially the cardiovascular system, hematologic system, and nervous system, and summarize characteristic biomarkers related to diagnosis, efficacy, and prognosis, aiming to provide assistance in clinical supervision and evaluation of treatment effectiveness (**Figure 1**; **Table 1**).

**Table 1:** Summary of symptoms, pathogenesis, incidence rate, potential biomarkers and monitoring methods of different systemic toxicity.

System	Common Symptom	Possible pathogenesis	Morbidity	Potential Biomarkers and Indicators				
				Prediction	Diagnostics		Severity	Prognosis
Cardiovascular System	arrhythmias, cardiovascular dysfunction, heart failure, cardiovascular death, acute coronary syndrome, cardiomyopathy, cardiac arrest, and other cardiovascular events	CRS induced by targeted therapy	13%-20.5%	cfDNA(ND1、mtCOX2 and Nkx2.5)	Cytokines (IL-6, IL-10, IFN-γ,et al.)		hs-cTnT	——
		off-tumor effects			Biochemical markers (CRP, ferritin, BNP, cTn, et al.)		NT-proBNP	
		off-target effects causing damage					Ang-1:Ang-2	
Blood System	fever, hepatosplenomegaly, cytopenia, liver dysfunction, elevated serum ferritin and transaminases, coagulopathy with hypofibrinogenemia, neurological abnormalities	CRS	2-36%(ICAHT)	CAR-HEMATOTOX	blood counts		blood counts	Cytokines (MDC、FGF-2、TGF-α、VEGF, MIP-1a and MIP-1b,et al.)
		lymphodepletion prior to CAR-T infusion	19%-69%(infection)		Cytokines (IL-6, IL-10,et al.)		Cytokines (IL-6, IL-10,et al.)	
					Biochemical markers (CRP, ferritin)		Biochemical markers (CRP, ferritin)	
		high tumor burden	-3.4%(IEC-HS)	Cytokines (IL-6, IFN-γ, granzyme B, IL-1RA and IL-10,et al.)	PT, APTT		Severity of CRS	occurrence time of cytopenia
		damage to the immune and hematopoietic systems	-56%(Coagulation disorder)		PCT			
Nervous System	tremors, writing disorders, mild speech difficulties (especially naming objects), and certain consciousness disorders, ataxia, delirium, seizures, and cerebral edema	activation of vascular endothelial cells	4.5%-65%	cfDNA	Blood	ferritin	GFAP、NfL(Blood, Cerebrospinal Fluid)	EASIX、mEASIX
						CPR		
						Cytokines(Special attention should be given to IL-6; IL-12, which may serve as markers for detecting independent ICANS)	Special amino acids(Amino acid hydroxyproline, Glutamine,et al.)	
					Cerebrospinal Fluid	Cytokines(IL6, IL8, MCP1, IP10, CD25, GM-CSF, et al)	[18F]FDG PET liver, spleen SUV level	
		breakdown of the blood-brain barrier						
		damage to central nervous system cells						
		infiltration of CAR-T						
Respiratory System	hypoxia, pneumomediastinum, pulmonary embolism, isolated pleural effusion, allergic rhinitis and respiratory failure	——	——	——	LDH		——	——
Digestive System	diarrhea, pancreatitis, constipation, perianal fistula, esophagitis, and vomiting	——	——	——	AST, ALT		——	——
					cfDNA derived from liver cells			
Urinary System	Acute renal failure and water-electrolyte imbalance	——	——	——	UA, CK		——	——

*All the incidence data have limitations as they are derived from the articles included in this paper. Due to the limited research on other systems (respiratory, digestive, and urinary), we did not include the limited data in our tables. All mentioned potential biomarkers and indicators are sourced from various studies; we merely present them as possible predictive indicators. The specific roles need further clinical research to be confirmed.

## 2 Cardiovascular system

### 2.1 Clinical feature

The association of cardiovascular risks with CAR-T cell therapy is a critical concern for patients. These risks may stem from a high tumor burden prior to treatment, the use of cardiotoxic anthracycline drugs, or pre-existing cardiovascular diseases in the patients. Consequently, patients with serious cardiovascular complications are typically deemed unfit for CAR-T therapy. In two major clinical trials of CAR-T for refractory DLBCL, patients with left ventricular ejection fractions (LVEF) below 45%-50% were excluded to minimize potential risks (13). A cross-sectional study including all adverse events(AEs) related to CAR-T reported by the FDA from 2017 to 2019 found that 19.7% of these events were cardiovascular in nature (14), consistent with the incidence rates of 13%-20.5% reported in other CAR-T studies (15–19). This data highlights the significant prevalence of cardiovascular issues following CAR-T therapy. A meta-analysis involving 2059 patients showed that following CAR-T treatment, the incidence of cardiovascular events such as arrhythmias, cardiovascular dysfunction, heart failure, cardiovascular death, acute coronary syndrome, cardiomyopathy, cardiac arrest, and other cardiovascular events were 19.2%, 8.0%, 5.3%, 1.8%, 2.5%, 2.9%, 1.3%, and 1.9% respectively (20), underscoring the need for vigilant monitoring and management of these risks.

#### 2.1.1 The impact of age on cardiovascular toxicity

While cardiovascular toxicity from CAR-T therapy is evident, it varies between children and adults. Two retrospective studies targeting children and adolescents indicate that CAR-T-related cardiac toxicity complications are mostly self-limiting, with the vast majority of patients’ cardiac functions gradually returning to baseline levels after treatment (21, 22). Research by Burstein et al. observed that persistent functional impairments were exceedingly rare among patients with cardiovascular toxicity over a six-month follow-up, with no cardiovascular adverse events leading to death (21). In contrast, in adults, cardiovascular toxicity manifests as a long-term issue. Lefebvre et al.’s study showed that in adult patients, the cumulative occurrence rates of major cardiac events 30 days, 6 months, and 12 months post-CART treatment were 17%, 19%, and 21% (23), respectively. This disparity suggests that adult patients may require more intensive and prolonged surveillance. Considering the limited sample size and brief follow-up duration, it is necessary to conduct studies with larger samples and extended follow-ups to ascertain the varying effects of CAR-T therapy on cardiovascular toxicity among children and adults.

#### 2.1.2 The impact of target spot on cardiovascular toxicity

Cardiovascular toxicity from CAR-T cell therapy shows little variation related to target antigens. In a single-center retrospective study on relapsed and refractory MM, 11 out of 78 patients (14.1%) treated with B-cell maturation antigen (BCMA)-targeted CAR-T therapy experienced cardiovascular AEs, a rate comparable to those observed in CD19-targeted CAR-T therapy. This finding indicates that CAR-T-induced cardiovascular toxicity is generally consistent regardless of the target antigen (17). Additionally, the severity of cardiovascular toxicity from CAR-T therapy is strongly linked to the severity of CRS. Lefebvre et al.’s study demonstrates that Grade 3 or 4 CRS is directly linked to major adverse cardiovascular events (MACE) (23). Moreover, a meta-analysis highlights that in patients with CRS of Grade ≥2, the combined incidence of cardiovascular events reached 25.6% and 14.2% respectively (20).

#### 2.1.3 Existing cardiovascular burden is not a contraindication for treatment

Although CAR-T therapy can lead to cardiovascular toxicity, it is not universally contraindicated for patients with severe existing cardiovascular conditions. A male patient with refractory MM and severe heart failure who received anti-BCMA CAR-T treatment not only survived but also showed significant improvement in cardiac function and hematologic recovery during a two-year follow-up, thus significantly boosting clinical confidence in CAR-T therapy (24). The clinical effectiveness and safety of this approach must be rigorously tested in future specialized clinical trials.

### 2.2 Potential mechanisms of cardiovascular adverse events

It is essential to recognize that the specific mechanisms underlying cardiovascular toxicity associated with CAR-T therapy are not fully understood yet. Before CAR-T therapy, a high tumor burden in patients may increase the risk of toxicity development; dose-limiting side effects often occur when using anthracycline drugs, potentially causing cardiac damage not only through the inhibition of topoisomerase II but also by the production of free radicals and reactive oxygen species (25); pre-existing cardiovascular diseases may further elevate the risk of cardiotoxicity following CAR-T therapy (26). Research indicates that myocardial strain is closely linked to adverse cardiac events (27), and these compounding factors could significantly elevate the probability of developing cardiovascular toxicity following CAR-T therapy. Current research on the mechanisms involved proposes three hypotheses: CRS induced by targeted therapy, off-tumor effects, and off-target effects causing damage (28).

#### 2.2.1 CRS

CRS is the most common systemic inflammatory response after CAR-T therapy, characterized by the release of large amounts of inflammatory cytokines such as IL-2, IL-6, IL-10, INF-γ, TNF-α, and C-reactive protein (CRP), along with the local recruitment of immune cells, which can induce a localized cytotoxic response. These inflammatory cytokines can not only induce hypotension, myocardial edema, and contractile dysfunction but can also activate prostaglandins, potentially leading to tachycardia, hypotension, and multi-organ failure (26, 29). In both cardiovascular disease and cardiovascular toxicity models following CAR-T therapy, inflammatory factors have been found to cause microvascular dysfunction by increasing the permeability of vascular endothelial cells, which subsequently leads to myocardial dysfunction (30, 31). In studies of cardiomyopathy models, large amounts of IL-6 can lead to abnormal myocardial electrical activity and arrhythmic tendencies by activating the gp130/STAT3 signaling pathway, thereby affecting normal cardiac electrical activity by regulating L-type calcium channels and delayed rectifier potassium currents in ventricular myocytes (32, 33). Additionally, cardiac ion channel remodeling induced by TNF-α signaling pathway activation has also been extensively studied in ischemic heart disease (34).

#### 2.2.2 Endothelial injury

In patients with severe CRS, the overactivation of vascular endothelia can also lead to endothelial dysfunction (18, 35). Clinical research data show that patients with severe CRS have higher concentrations of VWF and elevated serum Ang-2 levels (35), providing evidence of endothelial damage through these elevated markers of endothelial activation. The massive release of cytokines caused by CRS can lead to endothelial damage, while activated endothelial cells release cytokines and pro-coagulant mediators that further exacerbate vascular damage (36). Endothelial damage is considered an early event in the development of cardiovascular abnormalities (37), and the hypercoagulable state caused by endothelial damage may lead to the formation of microthrombi within blood vessels. These microthrombi can further burden the cardiovascular system and may cause ischemia and dysfunction in various bodily systems. Particularly in the setting of CRS, the excessive activation of the coagulation system significantly raises the risk of thrombosis. Therefore, to mitigate endothelial damage, the use of suitable cytokine antagonists, timely anticoagulation, antioxidant therapy, and endothelial cell protectants is necessary, particularly in conditions that might lead to thrombosis. Some articles suggest considering the use of statins (38, 39).

#### 2.2.3 Off-tumor effects and off-target effects

Cancer infiltration and excessive inflammatory responses in the tissues surrounding the heart can also directly affect the heart; invasion of the mediastinal lymph nodes by tumors provides a target for CAR-T cells, and the subsequent attack may cause local inflammatory responses due to the release of intracellular contents from lysed cancer cells, impacting cardiac function (31, 40); significant pericardial effusion caused by pericarditis can also physically compress the heart, thereby impairing its function. In a case report of cardiac tamponade induced by CAR-T cell therapy, 73% of CAR-T cells were found in the pericardial fluid during a recurrence (41), providing potential evidence for the role of CAR-T cells in cardiac toxicity.

### 2.3 Potential monitoring indicators for preventing toxicity

#### 2.3.1 Predictive indicators for the onset of toxicity

Circulating free DNA (cfDNA) refers to the free endogenous deoxyribonucleic acid (DNA) and exogenous DNA in the circulating blood (42). cfDNA has been confirmed to be useful for monitoring minimal residual disease and relapse after cancer treatment (43–45). In addition, in clinical studies of leukemia, it has also been shown to predict the prognosis of patients with acute leukemia (46). It is a biomarker in liquid biopsy that can be used for early diagnosis, prognosis, and monitoring of diseases (47), and is particularly valuable in hematologic diseases (48). The proliferation and killing of tumor cells by CAR-T cells produce a large amount of tumor cell debris. If organ toxicity occurs, the damaged organs also release a large amount of cell debris into peripheral blood, pleural and peritoneal fluids, cerebrospinal fluid, and bronchoalveolar lavage fluid in the form of free DNA or RNA. This debris, due to its unique physiological characteristics, allows for organ tracing to locate the damaged organ (49), effectively reflecting or predicting damaged organs or other toxic reactions during CAR-T cell treatment. Before fatal organ toxicity occurs during CAR-T cell immunotherapy, tracing the damaged tissues from plasma can facilitate timely interventional treatment for patients. Silver et al. studied the role of cfDNA in cardiotoxicity and found that the abundance of specific markers (ND1, mtCOX2, and Nkx2.5) in cfDNA might predict cardiotoxicity (45). Specific cfDNA in body fluids might also serve as a monitoring indicator for cardiotoxicity after CAR-T infusion, though its effectiveness needs further clinical research to be validated.

#### 2.3.2 Confirmatory indicators for the presence of toxicity

Cardiovascular injuries following CAR-T infusion are frequently observed against the backdrop of CRS, underscoring the importance of monitoring changes in corresponding biomarkers. Several studies have confirmed that during CRS, a variety of cytokines such as IFN-γ, IL-2, IL-6, GM-CSF, IL-1, IL-8, IL-10, IL-12, TNF-α, MCP-1, and MIP 1α exhibit fluctuations. Simultaneously, multiple biochemical indicators such as CRP, ferritin, and lactic dehydrogenase (LDH) also undergo significant changes (35, 50, 51). The alterations in IL-6, IL-10, and IFN-γ are pronounced and clinically relevant, with IL-6 playing a pivotal role by markedly enhancing the production of ferritin and CRP (52). According to Qi et al.’s research, patients with earlier peak concentrations of IL-6 and IFN-γ experienced higher levels of cardiac damage, as these cytokines can cause endothelial damage (18). In response to these challenges, tocilizumab, an IL-6 monoclonal antibody, is commonly used to treat CRS (53), and IFN-γ is being explored as a potential new target for heart disease caused by CRS or CAR-T therapy.

A clinical study involving patients undergoing CAR-T therapy revealed significant findings related to cardiovascular biomarkers. It showed that 163 patients (81%) had elevated CRP levels, 162 (80%) exhibited elevated ferritin levels, 61 (30%) had elevated IL-6 levels, 66 (33%) displayed elevated troponin levels, and 55 (27%) had elevated natriuretic peptide levels. Importantly, peak levels of IL-6, CRP, ferritin, and troponin were higher in those who experienced cardiovascular AEs. While elevated troponin levels are commonly seen in patients receiving CAR-T cell therapy, those who develop cardiovascular toxicity have a high incidence, with elevated troponin found in as many as 94% of cases (15). Both troponin and natriuretic peptides are frequently referenced in research focused on cardiovascular toxicity following CAR-T infusion.

Troponin, composed of the subunits troponin T (cTnT), troponin I (cTnI), and troponin C (cTnC), is a crucial biomarker for myocardial injury and necrosis, playing an essential role in diagnosing and stratifying the risk of acute myocardial infarction. During the early phase of myocardial injury, the rise in blood cTn levels is gradual, and traditional cTn testing methods initially show low sensitivity. To address this, advancements in diagnostic technologies have led to the development of high-sensitivity troponin (hs-cTn) testing, which offers improved sensitivity and accuracy for the early diagnosis of myocardial infarction (54). Natriuretic peptides (NPs), quantitative plasma biomarkers, indicate the presence and severity of hemodynamic cardiac stress and heart failure (HF). B-type natriuretic peptide (BNP), originating from the ventricles, is secreted by myocardial cells as a precursor that is cleaved by activating enzymes into N-terminal pro b-type natriuretic peptide (NT-proBNP) and BNP before being released into the bloodstream (55, 56). In a report by Alvi et al., of 13 cardiac patients following CAR-T infusion, 9 and 12 exhibited elevated high-sensitivity troponin T (hs-cTnT) and NT-proBNP, with median peak values at 23.60 ng/L and 3657 pg/ml respectively (57); Shalabi et al. reported increased pro-BNP levels during CRS, with 4 (31%) cases exhibiting abnormal troponin levels (22); Moriyama et al. described a case of cardiac tamponade after CAR-T infusion with increased serum troponin T (0.038 ng/mL; normal range: ≤0.014 ng/mL) and BNP (323.3 pg/mL; normal range: ≤18.4 pg/mL) (58).

#### 2.3.3 Assessment indicators for the severity of toxicity

Qi et al. observed a positive correlation between hs-cTnT levels and serum levels of IL-6, ferritin, and IFN-γ, while NT-proBNP levels positively correlated with serum levels of IL-6, ferritin, IFN-γ, and IL-10. They noted that the severity of cardiac conditions correlates with higher peak values of hs-cTnT, NT-proBNP, ferritin, CRP, and various cytokines including IL-6, IL-8, IFN-γ, and IL-10 (18). Both cTn and BNP have known peak times and durations in cardiac injury. Alvi et al. proposed that 95% of cardiovascular injury events after CAR-T therapy occur after an increase in troponin levels (57). Similarly, the increase in pro-BNP levels usually occurs during CRS and is significantly correlated with the severity of the condition. More frequent monitoring of these levels may help in the early identification of patients at risk for cardiovascular toxicity and in reducing cardiac toxicity through early CRS intervention. Additionally, the peak levels can be used to determine the severity in the midterm. Furthermore, Ghosh et al. proposed using troponin and NT-proBNP as markers for cardiac function risk assessment before administering CAR-T infusions (59).

Monitoring the levels of troponin, NT-proBNP, and characteristic cfDNA in patients prior to CAR-T infusion can serve as predictive markers for cardiotoxicity. Therefore, clinically, patients with high levels of these indicators should be closely monitored for cardiovascular responses before treatment, raising awareness of potential cardiovascular toxicity and providing individualized stratified management. After CAR-T infusion, monitoring various cytokines like IL-6 and IFN-γ, as well as troponin and NT-proBNP, is crucial to prevent the onset and progression of cardiovascular toxicity. In addition, further research is needed to understand the role of characteristic cfDNA in this context.

## 3 Blood system

### 3.1 Clinical feature

Hematologic toxicity is a common systemic toxicity observed in patients after CAR-T cell infusion, reported across various hematologic malignancies and regardless of the CAR-T treatment targets (60–70). The clinical presentation mainly includes Immune Effector Cell-Associated Hematotoxicity (ICAHT), Infection, Secondary Hemophagocytic Lymphohistiocytosis (sHLH), Graft-versus-Host Disease (GvHD) and Host-versus-Graft Disease(HvG), Coagulation Disorders.

#### 3.1.1 ICAHT

Post-CART therapy cytopenia is a common hematological toxicity, which has specific clinical manifestations, rendering the classification systems used for cytopenia after conventional cytotoxic chemotherapy unsuitable. To further describe and manage this adverse event, the European Hematology Association (EHA) and the European Society for Blood and Marrow Transplantation (EBMT) introduced the term ICAHT to define it, confirming its clinical relevance in real-world studies (71, 72). Immune Effector Cell-Associated Hematoxicity (ICAHT) is primarily characterized by severe cytopenia, including neutropenia, thrombocytopenia, and anemia, and is graded according to the severity and duration of neutropenia. Patients typically experience a transient period of bone marrow suppression after CAR-T therapy, during which hematopoietic function significantly declines, leading to a sharp decrease in blood cell counts. The risk model CAR-HEMATOTOX is utilized to identify patients at high risk for clinically significant cytopenia and assists in risk-adjusted management of hematotoxicity, encompassing hematopoietic reserves (ANC, hemoglobin, and platelet counts) and baseline inflammation (CRP and ferritin levels).

Thirty days post-CART infusion is regarded as the dividing line between early and late phases, with occurrences within 30 days defined as early ICANS. Classical ICAHT manifests as biphasic changes; lymphodepletion prior to infusion carries hematologic toxicity (73), early cytopenia appears within the first two weeks post-CART treatment, and late recurrence far exceeds the expected time related to lymphodepleting chemotherapy preparation (66), persisting as prolonged hematologic toxicity (PHT). Regarding lymphodepletion choices, clinical studies have proven that bendamustine, used as an alternative to the standard lymphocyte clearance regimen of fludarabine combined with cyclophosphamide (FC), exhibits lower hematological toxicity and reduced antibiotic use (74). Clinical trials have observed a high incidence of PHT (72.04%) one month after using CAR-T cells to treat patients with relapsed/refractory MM, manifested as single-lineage or multilineage reductions (68). CAR-T treatment of solid tumors shows similar patterns (75). Neutropenia is particularly prominent, with a meta-analysis indicating that the incidence rates of any-grade neutropenia, thrombocytopenia, and anemia are 80%, 61%, and 68%, respectively. The occurrence rates of Grade ≥3 neutropenia, thrombocytopenia, and anemia are respectively 60%, 33%, and 32% (76). An important note is that a decrease in neutrophils significantly raises the risk of infection, which will be elaborated on further below.

#### 3.1.2 Infection

Prolonged cytopenia can lead to a decrease in the body’s resistance, significantly increasing the chances of serious infections. A meta-analysis indicates that the infection rate in patients receiving CAR-T infusion is high (40%), with 25% being serious infection events (77). Regardless of the target, 19%-69% of patients reported the occurrence of infections (78, 79). Clinical research has suggested a link between severe cytopenia, high infection rates, and poor survival rates. Additionally, it has been found that severe infection is a high-risk factor for non-relapse mortality (63, 65, 70). A meta-analysis involving 7,604 patients indicated that 50.9% of non-relapse deaths were due to infections (80). Although prolonged cytopenia is a high-risk factor for infection, infections after CAR-T infusion are multifactorial and not solely due to cytopenia. For instance, lymphocyte depletion before CAR-T infusion can significantly reduce immunity, particularly if the patient has an existing infection before the lymphocyte depletion, which can lead to the spread of infection after treatment (81). Due to the different stages of target expression in B lymphocyte cells, targeting CD19 causes B cell impairment, while targeting BCMA leads to plasma cell depletion, resulting in different immunodeficiencies that may require various preventive measures (78). Other immunosuppressive factors are also risk factors for secondary infections (82).

Bacteria, viruses, and fungi can all be pathogens of infections after CAR-T infusion, with bacterial infections being the most common (81). Although early use of antibiotics is commonly practiced clinically to prevent infections, its role in post-CART infusion infections has not been clinically confirmed. Moreover, the EHA/EBMT recommends against the early use of antibiotics for prophylaxis in patients at low risk of ICAHT (71). Among patients who developed bacteremia after receiving CD19-targeted CAR-T therapy, 33% had Gram-negative bacteria with acquired or intrinsic fluoroquinolone resistance (81). For patients with confirmed secondary bacterial infections, early de-escalation and discontinuation of antibiotics can significantly reduce the use of broad-spectrum antibiotics without affecting CAR-T treatment outcomes (83). Among viral infections, respiratory viral infections are the most common. Cytomegalovirus (CMV) is also a concern due to its prevalence and the severe complications it can cause in immunocompromised patients. Although many studies focusing on CMV reactivation after CAR-T therapy have not reported terminal CMV organ disease (84–86), a case report showed a patient who achieved complete remission after anti-CD19/22 CAR-T cell therapy but was rehospitalized three months later due to CMV infection. Despite effective early treatment, symptoms became uncontrollable after the removal of ganciclovir, and the patient eventually died of respiratory failure (87). This suggests that although CMV infection after CAR-T infusion is usually considered self-limiting and not related to terminal organ disease, close monitoring for CMV reactivation is necessary for these special patients. JC polyomavirus (JCPyV), human herpesvirus 6 (HHV-6), herpes simplex virus (HSV), and varicella-zoster virus (VZV) can also be pathogens (88, 89). The EHA/EBMT recommends that all patients begin antiviral treatment with valaciclovir or aciclovir upon the initiation of lymphocyte depletion. Invasive fungal infections occur in 8% of patients, including mold infections (90). Clinical studies show that without antifungal prophylaxis, 8 out of 280 treated patients (2.9%) developed invasive fungal disease, including pneumocystis jirovecii pneumonia, invasive mold infections, and invasive yeast infections (91). Antifungal prophylaxis is usually applied in cases of severe neutropenia (absolute neutrophil count (ANC) < 500/μL) and high ICAHT risk.

#### 3.1.3 sHLH or IEC-HS

sHLH or Macrophage Activation Syndrome (MAS) is a rare but life-threatening hyperinflammatory syndrome that can be triggered by infections, malignancies, metabolic disorders, autoimmune diseases, and specific treatments. It can lead to fever, hepatosplenomegaly, cytopenia, liver dysfunction, elevated serum ferritin and transaminases, coagulopathy with hypofibrinogenemia, and neurological abnormalities (60). A study reviewing literature and practice within EBMT centers estimated the incidence of sHLH/MAS following CAR-T cell therapy at 3.48% (92). Song et al.’s research shows that although the incidence of hematologic toxicity of HLH after CAR-T infusion is low, HLH can lead to a mortality rate of up to 69.9% (93). CAR-T-related HLH rarely occurs without the background of CRS and is considered an overlapping syndrome with CRS and a variant of CRS (60, 94, 95). The CAR-T-cell-therapy-associated TOXicity (CARTOX) Working Group was the first to systematically propose that severe CRS can evolve into HLH (32). Clinical studies indicate that all cases of carHLH occur after the onset of CRS. Notably, the response of this syndrome to tocilizumab often differs from that of severe CRS and generally leads to worse outcomes (94, 96, 97). Case reports indicate the effectiveness of anakinra and ruxolitinib in achieving clinical remission (96). Cheng et al. proposed that a unique cytokine network can distinguish carHLH from CRS (98).

Increasing studies have observed the occurrence of secondary HLH after CAR-T therapy, which does not entirely overlap with CRS. To manage this more effectively, the American Society for Transplantation and Cellular Therapy (ASTCT) group has defined this syndrome caused by cell therapy as “Immune Effector Cell (IEC)-associated HLH-like syndrome (IEC-HS)” and proposed corresponding grading and management strategies (99). IEC-HS shares many similarities with CRS, but its diagnostic criteria are distinctly different. Fever is excluded from the diagnostic criteria of IEC-HS, with a particular emphasis on ferritin levels. The main criteria include levels twice the upper limit of normal, twice the ferritin level at the time of infusion, or rapidly rising ferritin. Classic first-line drugs for sHLH treatment usually include steroids (100). The new criteria add anakinra as a first-line drug for IEC-HS, and the IFN-γ inhibitor emapalumab has been shown to achieve clinical remission (101, 102). Theoretically, since IEC-HS patients often have elevated IL-18, its blocker Tadekinig alfa has been found to alleviate XIAP deficiency, a susceptibility condition for HLH, in clinical studies, which might be an effective drug yet to be discovered (103–105). Since IEC-HS has only recently been proposed, further clinical exploration is awaited.

#### 3.1.4 GvHD and HvG

Autologous CAR-T therapy requires T lymphocytes from the cancer patient’s own body. This process, spanning from production to treatment, is not only complex but also economically burdensome. Moreover, some immunocompromised patients cannot generate autologous T cells due to their underlying conditions. To address this issue, currently, developing allogeneic “off-the-shelf” CAR-T cell therapies is seen as a promising solution to overcome this limitation. However, due to the unique immune characteristics of graft-versus-host disease (GvHD), infused CAR-T cells may attack host tissues and be rejected by the host’s immune system (106). Consequently, acute GvHD may cause multiple organ damage and systemic inflammation, while chronic GvHD leads to chronic organ inflammation and fibrosis. In one notable instance, Yang et al. reported a case of a patient receiving unedited allogeneic CAR-T therapy who developed mild chronic GvHD of the skin and eyes (107). In response to these challenges, currently, various molecularly modified allogeneic CAR-T cells are under clinical investigation to enhance safety and efficacy.

#### 3.1.5 Coagulation disorders

Coagulation disorders can occur after CAR-T cell infusion. According to meta-analysis data, the incidences of prolonged activated partial thromboplastin time (APTT) and hypofibrinogenemia were 56% and 13%, respectively, while the incidences for ≥3 grade prolonged APTT and hypofibrinogenemia were 4% and 5%, respectively. Subsequent disease subgroup analysis studies found that the probability of coagulation disorders is similar across different disease types (76). The most severe coagulation disorder, disseminated intravascular coagulation (DIC), can be life-threatening (108). Research indicates that coagulation disorders are related to CRS and endothelial disruption, both of which can be factors in the pathogenesis of consumptive coagulopathy and DIC (109). Buechner et al. found that patients exhibited extremely low fibrinogen levels (<1g/L), prolonged prothrombin time, activated partial thromboplastin time, and increased international normalized ratio, all of which are prerequisites for coagulation disorders (60). Consequently, after CAR-T cell therapy, most patients’ coagulation disorders are manageable but still require close monitoring, early diagnosis, and timely intervention.

### 3.2 Potential mechanisms of hematological adverse events

#### 3.2.1 CRS

CRS may be closely related to the occurrence of hematologic toxicity, with numerous studies demonstrating that the severity of CRS correlates with early cytopenia, prolonged hematologic toxicity, coagulopathy, and IEC-HS (60, 62, 66, 70, 110, 111). During CRS, the monocyte/macrophage system releases a large amount of inflammatory factors, including IL-6, IL-1, ferritin, and INF-γ. Zhou et al. reported that early severe cytopenia correlate positively with the incidence and severity of CRS and peak levels of inflammatory markers (IL-6, CRP, and ferritin) (70). During grade 3 and 4 CRS, studies by Buechner et al. found elevated ferritin levels, prolonged PT and APTT, and decreased fibrinogen (60). Multiple studies have shown that IL-6 can influence megakaryocyte maturation and platelet release from the bone marrow, myeloid differentiation during neutropenia, T cell differentiation, and B cell antibody production (112–115), all factors significantly related to the production, maturation, differentiation, and death of blood cells.

#### 3.2.2 Other mechanisms

Early cytopenia are not only associated with CRS but are also thought to be related to lymphodepletion prior to CAR-T infusion, high tumor burden, and CAR-T cell-induced damage to the immune and hematopoietic systems (66). Preparatory lymphocyte depletion from chemotherapy before CAR-T cell infusion, resulting in cytopenia and subsequent infections, is among the most critical long-term adverse effects. A decline in white cells within the initial two weeks may relate to LD therapy, a period marked not just by reductions in mature myeloid or lymphoid cells but also in peripheral blood hematopoietic progenitor cells (73). Brudno and others have proposed that cytopenia in patients who haven’t received chemotherapy post-CART infusion suggest that CAR-T cells directly induce bone marrow suppression or destruction (116). Rejeski and others found that 64% of patients continue to experience neutropenia long after lymphocyte depletion and acute CRS have subsided (110). This observation supports persistent cytopenia lasting more than 30 days after CAR-T cell infusion suggest that their cause is unrelated to earlier lymphocyte depletion (117). This indicates that the biphasic pattern of cytopenia might stem from two different mechanisms. Late hematologic toxicity/PHT is thought to be related to bone marrow suppression induced by CAR-T cells, increased inflammatory factors, or the activation of an excessive immune response (35). A clinical study involving seven PHT patients receiving bone marrow biopsies post-CART therapy, all showing complete blood cell dysplasia or inadequate numbers of hematopoietic cells, suggesting evidence of bone marrow suppression by CAR-T cells (111). In a group of 38 patients who underwent CAR-T therapy, prior hematopoietic stem cell transplant (HSCT) was identified as a risk factor for hematologic toxicity (66), mostly occurring less than a year before CAR-T treatment, reflecting the overall intensity of treatment and incomplete recovery of the marrow, potentially leading to insufficient marrow function under hematopoietic stress (92, 118). Wang et al. noted that patients with a higher CD4/CD8 ratio in CAR-T cells had a lower incidence of long-term anemia, thrombocytopenia, and PHT, indicating that prolonged neutropenia might be driven by immune-related factors (68). Some paper links the inflammatory response after CAR-T therapy to its negative impact on hematopoietic stem cells. The researchers emphasize that the stability of mature blood cells requires the homeostasis of hematopoietic stem cells, but the presence of inflammation often disrupts this balance, reducing the number and impairing the function of hematopoietic stem cells, potentially leading to long-term reduction in blood cells following CAR-T treatment (117, 119).

In coagulation disorders, a study by Jiang et al. measured over a continuous month in 15 patients showed elevated levels of TF and Platelet And Endothelial Cell Adhesion Molecule-1(PECAM-1) in plasma (120), indicating their significant roles in vascular endothelial coagulation dysfunction. PECAM-1, crucially expressed at intercellular junctions of endothelial cells to sustain vascular integrity, when damaged (121), prompts overexpression of TF, initiating the extrinsic coagulation pathway (122). This culminates in systemic microvascular thrombosis, massive depletion of coagulation factors, subsequent hyperfibrinolysis, and manifests as microcirculatory issues and bleeding (38).

#### 3.2.3 IEC-HS

IEC-HS is an excessive inflammation associated with T cell and macrophage activation, typically accompanied by concurrent natural killer (NK) cell dysfunction, which can induce cytokine-mediated systemic toxic cascades (123), the inflammatory cytokine storm induced by CRS following CAR-T infusion resembles this excessive inflammatory manifestation. Its characteristics mainly manifest as features of macrophage activation/HLH, induced by immunotherapeutic cells, but the specific mechanisms underlying its occurrence remain incompletely understood, necessitating further research to analyze its intrinsic mechanisms.

### 3.3 Potential monitoring indicators for preventing toxicity

#### 3.3.1 Predictive indicators for the onset of toxicity

Most hematological toxicities occur in the context of CRS. In retrospective studies analyzing hematological toxicities, changes in key biomarkers associated with CRS are observed in hematological toxicities. Clinical studies have proposed biomarkers such as TNF-γ and IL-6 to predict severe CRS (35). When changes in these indicators are detected, vigilance is needed not only for severe CRS but also for hematological toxicities. Comprehensive serum proteomics analysis after CD19-targeted CAR-T therapy indicates that FMS-like tyrosine kinase 3 (FLT3) and mast cell immunoglobulin-like receptor 1 (MILR1) are pre-infusion predictive biomarkers for severe CRS (124).

Baseline ANC, hemoglobin concentration, baseline ferritin, IL-6, platelet count, and baseline CRP are independent risk factors for ≥ grade 3 cytopenia (67). Based on extensive clinical research and experience, the CAR-HEMATOTOX proposed by EHA/EBMT can early identify high-risk patients for cytopenia and provide appropriate management. This model has been confirmed in retrospective studies (71, 110), and the corresponding ICAHT grading shows a stronger ability to predict severe infections (72).

For biomarkers not included in CAR-HEMATOTOX but confirmed by clinical studies to be altered, perturbations in stromal cell-derived factor (SDF-1) levels may be associated with late-onset neutropenia, and high levels of IL-10 and IL-17A are risk factors (66). In comprehensive analyses of long-term anemia, higher IL-6 levels and high baseline β2-microglobulin levels are independent risk factors for prolonged anemia, while high baseline hemoglobin is an independent protective factor. Baseline platelet count, high CD4/CD8 CAR-T cell ratio, and high baseline IL-2 levels significantly influence long-term thrombocytopenia levels (68).

IEC-HS typically causes clinical presentations and biomarker changes similar to those of CRS, so cytokines are rarely considered in the prediction and diagnosis of IEC-HS. Identifying cytokines that independently reflect IEC-HS separate from CRS could provide new strategies for clinical management. Zu et al. found through research that extreme elevations in IFN-γ, granzyme B, IL-1RA, and IL-10 can distinguish this condition. Based on this, they proposed a predictive model specifically for CAR-T therapy-related HLH and suggested that therapies targeting IL-10 could be a potential treatment approach (98).

#### 3.3.2 Confirmatory indicators for the presence of toxicity

Cytokines associated with the occurrence of CRS can also be used to determine the occurrence of hematological toxicity. Early severe cytopenia are related to the severity of CRS and the peak levels of inflammatory factors (IL-6, C-reactive protein (CRP), and ferritin) (70). Case reports also provide corresponding evidence; patients with pancytopenia show significantly elevated levels of IL-10, ferritin, CRP, and IL-6 (122). Retrospective analysis reports that the higher the serum IL-6 level, the lower the endpoint blood cell count, while transforming growth factor (TGF)-β1 shows the opposite trend; the higher its serum concentration, the higher the blood cell count (62). Corresponding cytokines can serve as unique biomarkers to provide a new approach for clinically determining the occurrence of ICAHT. Serum ferritin, as a marker that increases in both CRS and IEC-HS, is recognized for its more distinctive changes in IEC-HS by an expert panel as diagnostic criteria for that (71).

When coagulation disorders occur, elevated levels of IL-6, CRP, and ferritin are positively correlated with coagulation indicators to a certain extent (108). The study by Buechner et al. emphasizes the relationship between coagulation dysfunction and the occurrence of CRS. They proposed that prothrombin time (PT) and APTT are prolonged shortly after CRS occurs, with fibrinogen levels initially increasing at the onset of CRS, and then decreasing during peak CRS and CRS resolution, often to very low levels (60). Fibrinogen is an acute-phase reactant, and when its serum level is low, bleeding events are highly likely to occur. Therefore, serum fibrinogen levels in patients who may develop coagulation disorders should be closely monitored. Coagulation-related indicators also need to be closely monitored by clinicians to prevent irreversible coagulation disorders in patients.

Fever often occurs after CAR-T infusion, and this fever usually cannot be clearly diagnosed early, such as whether it is due to CRS or accompanying infection. The study by Powell et al. suggested that an increase in serum procalcitonin (PCT) during the first fever after CAR-T infusion is significantly associated with confirmed bacterial infection (125). Confirmed infections usually require microbiological confirmation, which often takes valuable time. Finding rapid and effective biomarkers for diagnosing infections after CAR-T infusion is essential. Since this is only a single-center study and only focuses on bacterial infections, more research is needed to explore further.

#### 3.3.3 Assessment indicators for the severity of toxicity

Multicenter observational studies suggest that the cumulative duration of severe neutropenia is strongly positively correlated with the severity of ICAHT, and a reduction in multilineage blood cells is observed in patients with severe ICAHT. The incidence of severe infections in patients with severe ICAHT significantly increases, with a higher occurrence of life-threatening and fatal infections (72). This suggests that in the event of ICAHT, tracking fluctuations in various blood cell counts over time is meaningful for monitoring changes in severity, facilitating clinical supervision and management. Grade 3 or higher thrombocytopenia is significantly associated with Eastern Cooperative Oncology Group (ECOG) scores, baseline hemoglobin and platelet counts, baseline IL-6 and ferritin, baseline CRP, and CRS severity (67). The incidence and severity of coagulation disorders are positively correlated with CRS grade (108). Serum LDH levels are significantly elevated in the severe ICAHT group (72), but there are no related retrospective studies focusing on this, and further research is needed to confirm its role.

#### 3.3.4 Prognostic indicators related to outcomes

A study comparing cytokine concentrations between patients with complete blood cell recovery and those without complete recovery one month after CAR-T infusion found significantly increased concentrations of macrophage-derived chemokine (MDC), fibroblast growth factor-2 (FGF-2), TGF-α, vascular endothelial growth factor (VEGF), and chemokines [macrophage inflammatory protein-1a (MIP-1a) and MIP-1b] in the serum of patients with blood cell recovery (126). Multivariate analysis showed that baseline ANC <2.29×10^9/l, baseline hemoglobin <114.5g/l, and baseline IL-6 >21.43pg/l were independent factors influencing hematologic recovery (67). Regarding thrombocytopenia, bone marrow reserve before lymphocyte depletion and inflammation-related factors (logarithm of ferritin levels before CAR-T cell infusion and logarithm of peak ferritin levels after infusion) are associated with recovery time (127). Monitoring relevant cytokines after infusion helps predict the extent and time of recovery in patients with hematologic toxicity.

Survival studies targeting CD19 CAR-T patients found that patients with classical biphasic neutropenia exhibited the best survival outcomes, which may be related to the higher CAR T cell expansion and persistence detected in these patients (82). More and longer-lasting CAR-T cells can bring more significant effects. The occurrence time of cytopenia can provide new insights into understanding survival differences.

Biomarkers such as TNF-γ and IL-6 not only predict CRS but also indicate hematologic toxicity. Baseline ANC, hemoglobin, ferritin, IL-6, platelet count, and CRP are recognized as independent risk factors for severe cytopenia. The CAR-HEMATOTOX scoring system effectively identifies high-risk patients. Other markers such as SDF-1, IL-10, and IL-17A are associated with neutropenia and prolonged anemia. Elevated serum PCT is associated with bacterial infection. Patients with severe ICAHT face a higher risk of infection and multilineage cytopenia. Baseline ANC, hemoglobin, and IL-6 levels are key independent predictors of hematologic recovery, and serum LDH is significantly elevated in patients with severe ICAHT. Additionally, occurrence time of cytopenia helps predict patient prognosis.

## 4 Nervous system

### 4.1 Clinical feature

Patients receiving CAR-T infusions often exhibit ICANS, which the American Society for Transplantation and Cellular Therapy (ASTCT) defines as a potentially progressive central nervous system pathology caused by immune activation in patients undergoing immunotherapy (12). Clinically, ICANS manifests from mild symptoms such as decreased attention, dysgraphia, aphasia, and cognitive impairment, to severe central nervous system symptoms like ataxia, delirium, seizures, and cerebral edema (12, 65, 128). Some symptoms, such as headaches, tremors, myoclonic jerks, asterixis, and hallucinations, are typically not categorized under ICANS due to their non-specific nature and using the conventional symptomatic treatment rather than specific intervention therapies (12). Yakoub-Agha et al. proposed that due to the early characteristic of ICANS being sloppy handwriting, daily handwriting tests in the months following CAR-T infusion can predict the onset of ICANS (129). ICANS is recognized as one of the most common adverse reactions to CAR-T therapy, with an incidence second only to CRS. A meta-analysis incorporating 42 hematologic malignancy studies, and 18 solid tumor studies showed that approximately 37.2% of patients with hematologic malignancies experienced varying levels of ICANS, while the incidence of CRS reached 55.3%. Notably, every CAR-T product demonstrating substantial clinical effectiveness has been linked to neurological adverse reactions, including targets like GPRC5D, CD7, NKG2D, CD19, and BCMA in different hematologic malignancies (8, 130–144) (**Table 2**).

**Table 2:** Summary of the incidence of CRS and ICANS in patients with hematological malignant tumors (partial data).

Target antigen	Trial-Registration	Proper Name	Disease	Phase	Case	CRS: All grades	CRS(3/4)	ICANS: All grades	ICANS(3/4)	Time	Reference
GPRC5D	NCT04555551	——	MM	I	17	15 (88%)	1 (6%)	1 (6%)	1 (6%)	2022	(130)
	NCT05016778		(R/R)MM	I	10	10 (100%)	0	0	0	2023	(132)
	ChiCTR2100048888		(R/R)MM	II	33	25 (76%)	0	2 (6%)	1 (3%)	2023	(131)
CD7	ChiCTR2000034762	——	r/r T-ALL	I	20	18 (90%)	2 (10%)	3 (15)	0	2021	(133)
	NCT04004637		r/rT-ALL/LBL(6) T-ALL(1) MPAL(1)	I	8	7 (87.5%)	1	0	0	2022	(8)
	NCT04538599		T-ALL (7)TCL(4)AML(1)	I	12	10 (83%)	0	0	0	2022	(134)
	NCT04572308		r/r T-ALL(14) T-LBL(6)	I	20	18 (90%)	1	2	0	2022	(135)
NKG2D	NCT02203825	——	MM(5) AML(7)	I	12	0	0	0	0	2019	(137)
	NCT03018405		MM(3)AML(12)MDS(1)	I	16	12 (75%)	5 (31.25%)	——	——	2023	(136)
CD19	NCT03575351	lisocabtagene maraleucel	DLBCL	III	92	45 (49%)	1 (1%)	11 (12%)	4 (4%)	2022	(138)
	NCT03331198		CLL/SLL	I-II	117	99 (85%)	10 (9%)	53 (45%)	22 (19%)	2023	(139)
	NCT02435849	tisagenlecleucel	R/R B-ALL	II	79	61 (77%)	38 (49%)	31 (39)	10 (13)	2023	(140)
	NCT04531046	Axicabtagene ciloleucel	R/R LBCL	II	62	58 (93.5%)	5 (8.1%)	32 (51.6)	9 (14.5)	2023	(142)
	——	brexucabtagene autoleucel	MCL	II	26	25 (96%)	2 (7.7%)	17 (65%)	10 (38%)	2024	(141)
BCMA	NCT02658929	Idecabtagene vicleucel	(R/R)MM	I	62	47 (75.8%)	4 (6.5%)	23 (37.1%)	1 (1.6%)	2023	(143)
	NCT04181827	ciltacabtagene autoleucel	(R/R)MM	III	176	134 (76.1%)	18 (1.1%)	8 (4.5%)	0	2023	(144)

*GPRC5D, G protein,coupled receptor class C group 5 member D; NKG2D , natural,killer group 2, member D; BCMA, B-cell maturation antigen; MM, multiple myeloma; R/R, relapsed/refractory; T-ALL, T cell acute lymphoblastic leukemia; LBL, lymphoblastic lymphoma; MPAL, mixed phenotype acute leukemia; AML, acute myeloid leukemia; MDS, myelodysplastic syndromes; DLBCL, diffuse large B-cell lymphoma; CLL/SLL, chronic lymphocytic leukemia/small lymphocytic lymphoma; MCL, mantle cell lymphoma.

#### 4.1.1 Relationship with CRS

The development of ICANS may be significantly correlated with the occurrence of CRS, with neurological adverse reactions typically occurring after CRS. According to Santomasso et al., all patients with ICANS had experienced at least grade 1 CRS before any neurological symptoms appeared, and some CRS treatments, like corticosteroids, are effective in managing ICANS, which suggests possible shared mechanisms (145). ICANS episodes are typically monophasic, with the typically median onset time of 7-9 days, often after CRS. Evidence from many studies indicates that fever often precedes ICANS; thus, monitoring for fever post-CART infusion is crucial for early detection of potential ICANS onset (65, 128, 145–147).

#### 4.1.2 Inspection results

In patients with ICANS, EEG typically shows generalized slowing of background activity (usually manifested as an increase in δ waves and θ waves), abnormal rhythms, and periodic discharge patterns (148). This condition may also present as diffuse slowing, epileptiform discharges (in cases where patients experience seizures or have underlying epileptiform activity), and non-convulsive status epilepticus (persistent seizure activity without obvious convulsive symptoms). Additionally, focal EEG abnormalities may present as lateralized periodic discharges (stimulation or damage to a specific area of one hemisphere), lateralized rhythmic delta activity (sustained abnormal rhythmic activity in one hemisphere), or focal slowing (149, 150). MRI is typically normal in the majority of ICANS patients (148). However, a study by Santomasso et al. indicated that a minority of patients may exhibit T2/FLAIR hyperintensities on MRI, involving the bilateral thalami and brainstem (145). Similarly, Gust et al. reported that MRI could show patchy white matter interstitial edema, T2 periventricular white matter, medullary, and/or thalamic hyperintensities (128). These imaging findings suggest that ICANS patients may experience different types of brain abnormalities throughout the disease course. Given that ICANS may be accompanied by brain imaging changes, regular monitoring with MRI and EEG is necessary for the surveillance of ICANS, differential diagnosis, and the occurrence of serious complications such as seizures and cerebral edema (151). These comprehensive clinical and imaging assessments help in the early detection and management of ICANS, thereby improving patient outcomes.

#### 4.1.3 Possible intervention

Preclinical studies have shown that IL-1 plays a role in the development of CRS/ICANS and demonstrated that the IL-1 antagonist anakinra can significantly reduce the severity of CRS/ICANS without affecting CAR-T cell activity (152). A single-center study reported that using anakinra in combination with other treatments in 14 patients who developed steroid-resistant ICANS, with or without CRS, following CAR-T infusion, has shown that anakinra might be a useful adjunct treatment to steroids and tocilizumab (153). Park and others further explored the effect of anakinra on ICANS in a Phase II clinical study, finding that prophylactic subcutaneous injections of anakinra at increased dosages could reduce the incidence of high-grade ICANS (154), which supports the idea that prophylactic administration of anakinra post-CART infusion could decrease the likelihood of severe and high-grade ICANS.

### 4.2 Potential mechanisms of neurological adverse events

The precise biological pathways of ICANS remain underexplored. Studies indicate that factors such as previous neurological compromise, ALL, pre-CART infusion tumor load, lymphodepletion strategies, elevated CD19^+^ cell counts in bone marrow, high CAR-T cell doses, and the severity of CRS all contribute to elevating the risk of ICANS onset (52, 129, 146, 151). CRS usually occurs before ICANS, and Gust et al. suggest that early episodes of CRS following CAR-T cell infusion are strongly correlated with the subsequent risk of severe neurotoxicity (146), possibly linked to the cytokine storm and infiltration of activated immune cells that occur during CRS.

The current understanding of the mechanisms behind ICANS largely involves the activation of vascular endothelial cells, the breakdown of the blood-brain barrier, damage to central nervous system cells, and the infiltration of CAR-T cells (52, 155).

#### 4.2.1 Activation of vascular endothelial cells

Studies indicate that in cases of ICANS, particularly in fatalities, there is significant activation of macrophages/microglia (CD68^+^), and pathological examinations typically reveal lymphocytes, CAR-T cells, macrophages, and signs of endothelial damage (156). Multiple studies have shown that in patients who developed ICANS, the ratio of Ang-2 to Ang-1 and the concentration of the von Willebrand factor (VWF) are elevated, primarily attributed to reduced levels of Ang-1 (145, 146). Ang-1 and Ang-2 counterbalance each other in maintaining endothelial stability; under normal physiological conditions, Ang-1 tends to stabilize endothelial cells and is typically more concentrated in the blood. Ang-1, through its binding to the Tie2 receptor, activates the PI3K/AKT pathway, which in turn promotes the accumulation of vascular endothelial cadherin, thus reducing endothelial permeability (157–159). Meanwhile, VWF regulates angiogenesis and interacts with Ang-2 (160, 161), providing substantial evidence that endothelial activation may be a risk factor for ICANS.

#### 4.2.2 Breakdown of the blood-brain barrier

**Figure 2:**
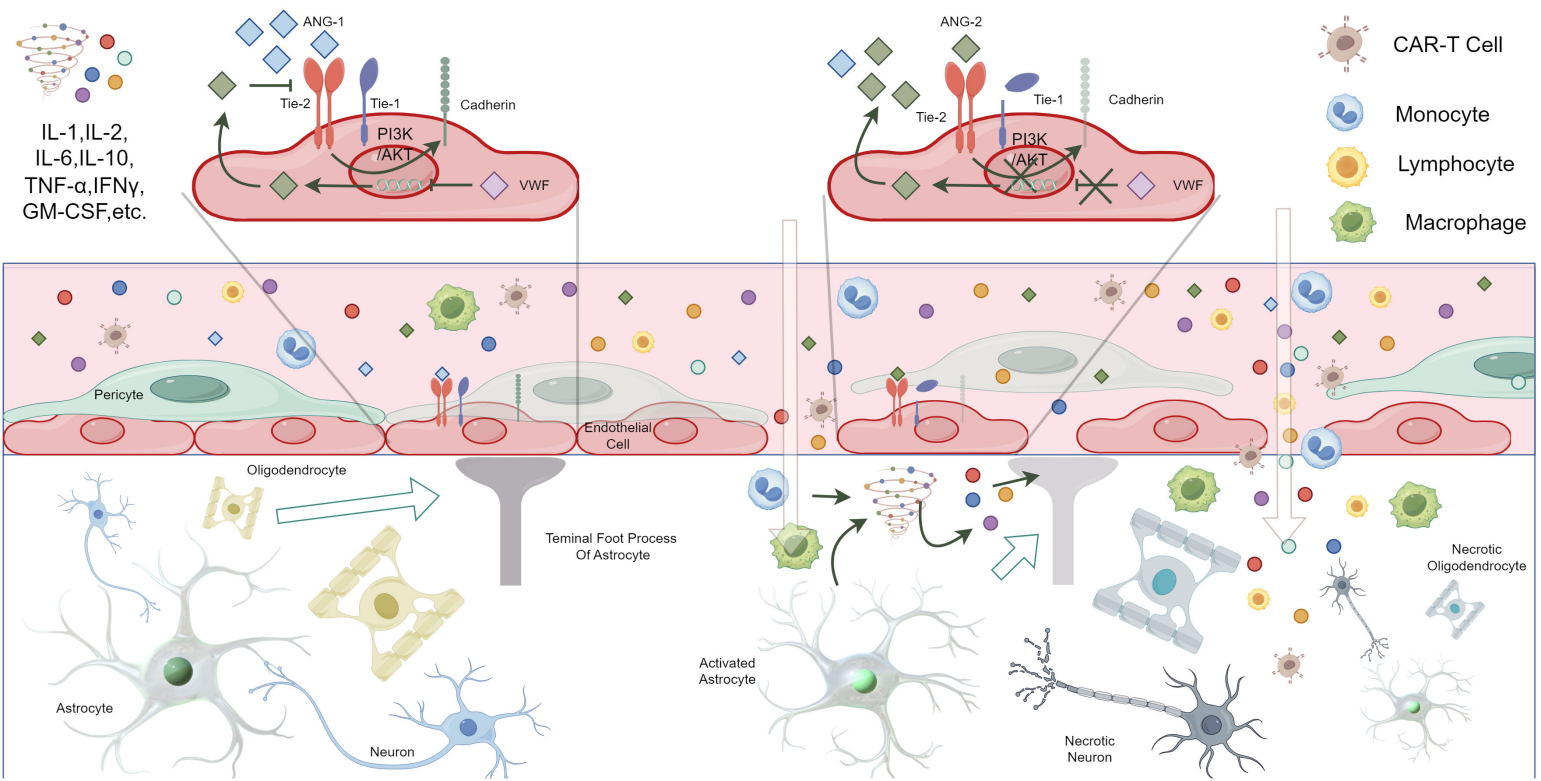
Possible pathogenesis of ICANS due to the activation of vascular endothelial cells, the breakdown of the blood-brain barrier, damage to central nervous system cells, and the infiltration of CAR-T cells (By Figdraw, ID: IPSUYeadf7). *Under normal conditions, Ang-1 and Ang-2 in the blood are in balance, with Ang-1 being more concentrated in the blood. Ang-1 binds to the Tie2 receptor on vascular endothelial cells, activating the PI3K/AKT pathway, which leads to increased expression of cadherin, maintaining tight junctions in the vascular endothelium and reducing permeability. Additionally, VWF inhibits the expression of Ang-2 by endothelial cells. Ang-2 competitively inhibits the binding of Ang-1 to Tie2, thereby increasing endothelial permeability. In ICANS patients, the level of Ang-1 decreases, leading to Ang-2 binding with Tie2, which reduces the production of cadherin and promotes the cleavage of auxiliary Tie-1, increasing endothelial permeability. This change, combined with the release of large amounts of pro-inflammatory cytokines and immune cell infiltration, results in the disruption of the blood-brain barrier. Consequently, a large number of cytokines and immune cells enter the central nervous system. The activation of astrocytes and microglia also leads to the massive release of cytokines, collectively attacking the central nervous system, causing central inflammation or parenchymal damage.

Activation of endothelial cells leads to changes in intercellular connections and matrix adhesion, and the activation of astrocytes and microglia that constitute the blood-brain barrier results in the abnormal release of pro-inflammatory mediators and the infiltration of immune cells, producing various cytokines that affect the permeability of the blood-brain barrier and lead to its disruption (162). Increased permeability of the blood-brain barrier allows for an influx of cytokines and CAR-T immune cells into the central nervous system through the blood-brain barrier, caused by CAR-T infusion. This causes severe central inflammation and brain parenchyma damage, leading to more severe and harder to treat central nervous system damage (163–165) (**Figure 2**). DelDuca et al.’s review indicates that lethal cerebral edema cases following CAR-T infusion were observed to exhibit neuronal death, neuronal edema, perivascular edema, and hemorrhagic extravasation within perivascular spaces and the parenchyma, further proving the significance of blood-brain barrier disruption in ICANS (156).

### 4.3 Potential monitoring indicators for preventing toxicity

#### 4.3.1 Predictive indicators for the onset of toxicity

Mika et al. analyzed and demonstrated the feasibility of using cfDNA to quantify CAR-T cells (166). The efficacy of CAR-T therapy depends on the number of amplified cells and their persistence. Wittibschlager et al. demonstrated that patients with persistent CAR-T cells had significantly higher CAR-T cell peaks and a higher incidence of ICANS (167). Therefore, it is speculated that cfDNA might serve as a predictive marker for ICANS, although specific effects need further clinical trials to confirm.

#### 4.3.2 Confirmatory indicators for the presence of toxicity

A clinical trial involving 25 patients reported increased serum ferritin and C-reactive protein levels in most patients who developed ICANS (147). Another study on pediatric CD19-targeted therapy found elevated levels of IFN-γ, IL-10, granzyme B, GM-CSF, MIP1α, and TNF-α in the serum of patients who developed ICANS (128). As the onset of ICANS typically accompanies CRS, it often results in significant alterations in serum cytokines thus challenging the independent analysis of ICANS-specific cytokines.

In their study, Gofshteyn et al. specifically focused on cytokines in the serum of ICANS patients independent of CRS, finding elevated levels of IL-2, IL-15, soluble IL-4, and hepatocyte growth factor compared to patients with isolated CRS, with notable differences in peak levels of IL-12 and soluble TNF receptor-1 within the first three days in ICANS patients (168), potentially enabling feasible detection of ICANS occurrence. The ratio of Ang-2 to Ang-1 in serum, along with VWF levels, has been identified as biomarkers for ICANS monitoring.

The unique pathogenesis of ICANS lends specificity and high operability to the detection of biomarkers in cerebrospinal fluid. The significantly increased permeability of the blood-brain barrier in ICANS patients enables a large influx of cytokines into the cerebrospinal fluid, thereby facilitating the monitoring of biomarkers therein. Several clinical investigations have corroborated this finding, as demonstrated in a study by Santomasso and colleagues, which noted elevated levels of IL-6, IL-8, MCP-1, and IP-10 during intense episodes of neurotoxicity (145). Frigault et al. also found increased levels of IL-6, CD25, and GM-CSF during peak toxicity periods (169). Monitoring cytokine concentrations in the cerebrospinal fluid of patients receiving CAR-T infusions can provide new insights for the clinical assessment of ICANS occurrence and the administration of specific immunotherapies.

#### 4.3.3 Assessment indicators for the severity of toxicity

Due to frequent injuries to the blood-brain barrier, such as astrocyte damage and central nervous tissue damage in ICANS patients, many researchers have focused on monitoring specific biomarkers of this damage, namely glial fibrillary acidic protein (GFAP) and neurofilament light chain (NfL). Neurofilaments (NF) maintain the mechanical stability of the axonal cytoskeleton. Axonal injury leads to the degradation of NFs, causing NfL to accumulate in the interstitial fluid of the brain, which is then released into the cerebrospinal fluid and blood (170). NfL levels has been proposed to be elevated in acute neuroinflammatory diseases (171). Similarly, GFAP, as an intermediate filament of the cytoskeleton, supports the interactions between astrocytes and other cells in the nervous system (172). Injury and degeneration of astrocytes lead to elevated GFAP levels in the cerebrospinal fluid and blood (173). High serum NfL levels prior to CAR-T infusion are significantly positively correlated with the incidence and severity of ICANS, with serum NfL levels remaining above baseline throughout the course of ICANS, and higher levels correlating with more severe ICANS classifications (174, 175). The cerebrospinal fluid also shows similar changes; although Gust and colleagues in their pediatric study found that baseline levels of GFAP and NfL in CSF are not predictive of ICANS (176), they can still serve as useful biomarkers for assessing the severity of CNS damage in patients who develop ICANS.

An analysis of metabolic products in patients before CAR-T infusion found that low concentrations of the amino acid hydroxyproline are positively correlated with higher grades of ICANS, and lower glutamine levels slow the progression of ICANS, with various metabolic differences also related to the development of CRS (177), suggesting that analyzing patients’ metabolomics before CAR-T infusion can help predict the risk of CRS/ICANS onset and assess the possible severity of the condition

[18F]FDG, a fluorodeoxyglucose injection, facilitates glucose metabolism in diseased organs and can be used with PET and CT imaging to detect diseases. Marchal et al.’s research found that a liver SUV mean >2.5 is associated with grade 2 to 4 CRS, and a spleen SUV average >1.9 is associated with grade 2 to 4 ICANS (178), highlighting these imaging features as helpful in clinical workflow for patient management.

#### 4.3.4 Prognostic indicators related to outcomes

The classical Endothelial Activation and Stress Index (EASIX) is defined as LDH × creatinine/platelets and the modified EASIX score (mEASIX) replaces creatinine with CRP to predict poor outcomes of CRS/ICANS associated with CAR-T infusion (179, 180).

Specific metabolite profiles in serum and NfL levels might become predictive markers for the occurrence and severity of ICANS prior to CAR-T infusion. Moreover, IL-2, IL-15, soluble IL-4, and IL-12 might act as independent monitoring factors for neurotoxicity development, apart from CRS. Additionally, cfDNA offers a new perspective for clinical monitoring. Due to the unique characteristics of ICANS, cerebrospinal fluid examination has become its most distinctive monitoring method. The measurement of specific inflammatory factors, especially the abundance of IL-6, provides reliable objective evidence for assessing the severity of ICANS. Meanwhile, GFAP and NfL are also potential monitoring indicators, but their reliability requires further clinical trials to validate. The appropriate imaging modalities such as MRI, [18F]FDG PET/CT, etc. are also an indispensable part of ICANS monitoring. Non-invasive examinations are more convenient and can enhance patient compliance.

## 5 Other systems

CAR-T-related toxicity affects multiple systems, including the respiratory, digestive, and urinary systems (59). Fusaroli et al., analyzing the FDA Adverse Event Reporting System (FAERS), noted the possible occurrence of gastrointestinal perforations, hepatotoxicity, and consciousness disturbances (181). Additionally, several studies have reported skin system damage following CAR-T infusion (182–184).

In patients with DLBCL treated with CD19-targeted CAR-T therapy, the incidence of grade 3 or higher pulmonary toxicity was 13.3%, with hypoxia being the most common complication, typically occurring within 10 days post-infusion. Furthermore, elevated LDH levels were associated with a higher risk of ≥ grade 3 pulmonary toxicity (185). LDH is considered a valuable biomarker for monitoring COVID-19 (186) and may also play a role in monitoring pulmonary toxicity in CAR-T therapies. CAR-T therapies targeting mesothelin (MSLN) for solid tumors have also been associated with lung damage. Haas and colleagues documented two instances of progressive hypoxemia occurring within 48 hours of CAR-T infusion, one of which escalated to grade 5 respiratory failure (187).

Liver toxicity is also common; Wudhikarn et al. observed 19 hepatic adverse events in 15 patients, typically involving elevated liver enzymes (185). Rates of increases in AST and ALT of any grade were observed at 28% and 29%, respectively, while significant increases (≥ grade 3) occurred at rates of 6% for AST and 2% for ALT (76). The measurement of hepatocyte-derived cfDNA is viewed as providing specific and sensitive information about hepatocyte death (188), making it a valuable prognostic criterion. Gastrointestinal toxicity often manifests as diarrhea, pancreatitis, and constipation, and may also include perianal fistulas, esophagitis, and vomiting (185).

Gérard et al. conducted a large-scale analysis supported by real data on renal safety, suggesting that acute renal failure (ARF) and a series of electrolyte imbalances are potential adverse drug reactions of CAR-T cell therapy (189). Research from Gupta and colleagues (190) also supports this perspective. Acute kidney injury following CAR-T infusion often shows a low incidence (17%), quick recovery (71% of AKI cases resolve within one month of infusion), and is significantly correlated with higher levels of uric acid (UA) and creatine kinase (CK) peaks, as well as changes in LDH from baseline to peak within one month post-treatment, significantly correlating with the incidence of AKI (191).

## 6 Conclusion and outlook

Tumor immunotherapy has become a continuous research focus, and CAR-T therapy is emerging as a significant option for patients with relapsed/refractory tumors, demonstrating astonishing therapeutic effects. The high incidence of adverse events following treatment necessitates the stringent monitoring and management of associated toxicities. Researching new biomarkers and searching for specific monitoring indicators can help detect early signs of toxicity and the severity of toxicities, enhancing clinical management and offering personalized medical services to patients. As CAR-T progresses in treating both hematological and solid tumors, toxicity monitoring will play a key role in enhancing the safety of CAR-T treatments in cancer patients and maximizing their therapeutic potential.
